# Egg-based nutrient delivery system: advances in omega-3, antioxidant, and micronutrient enrichment

**DOI:** 10.3389/fnut.2026.1770381

**Published:** 2026-01-22

**Authors:** Cui Ma, Md. Abul Kalam Azad, Xiaoyue Yu, Jing Sun, Hao Zhang, Jinsong Pi, Yan Wu

**Affiliations:** 1 Institute of Animal Husbandry and Veterinary Science, Hubei Academy of Agricultural Sciences, Wuhan, China; 2 Hubei Key Laboratory of Animal Embryo Engineering and Molecular Breeding, Wuhan, China; 3 Hunan Provincial Key Laboratory of Animal Nutritional Physiology and Metabolic Process, Institute of Subtropical Agriculture, Chinese Academy of Sciences, Changsha, China

**Keywords:** antioxidants, egg-based nutrient delivery, functional foods, micronutrients, omega-3 fatty acids

## Abstract

Eggs are nutrient-dense, animal-derived foods with significant health-promoting properties. Beyond providing high-quality protein, their unique structure and balanced nutrient matrix make them ideal carriers for omega-3 polyunsaturated fatty acids, antioxidants, and essential micronutrients. This review summarizes current strategies for egg fortification, focusing on nutrient deposition mechanisms, bioavailability, and functional outcomes. We integrate metabolic transport pathways, including intestinal absorption, hepatic processing, lipoprotein assembly, and yolk deposition, to elucidate how nutrients are efficiently incorporated. This work demonstrates how targeted enrichment can enhance egg nutritional value and support preventive nutrition. Understanding nutrient incorporation provides practical guidance for poultry management, feed formulation, and the production of functional eggs, positioning them as a natural and effective dietary delivery system. By adopting a mechanistic and strategy-oriented perspective, we highlight the potential of next-generation functional eggs as effective vehicles for improving nutrient intake and advancing preventive and precision nutrition.

## 1 Introduction

Eggs are widely consumed, cost-effective sources of high-quality, easily digestible protein and provide a balanced array of lipids, carbohydrates, vitamins, and minerals, making them efficient carriers of essential nutrients. Globally, egg consumption has steadily increased due to population growth and changing dietary habits (1), with China contributing nearly 40% of production (2). Beyond their nutritional value, eggs possess multifunctional properties: the yolk and albumen support emulsification, foaming, gel formation, and flavor delivery, making them indispensable in culinary and industrial applications. While most eggs worldwide are consumed as shell eggs (~70%), this proportion exceeds 95% in China (3). Consumer preferences for shell color, yolk pigmentation, and egg size vary across regions, reflecting the diversity of the global egg market.

Structurally, chicken eggs comprise the yolk, albumen, shell membranes, and shell, each with distinct biochemical characteristics. The yolk is particularly nutrient-dense, containing triacylglycerols, phospholipids, lipoproteins, and a variety of fatty acids, as well as micronutrients such as fat-soluble vitamins, B-complex vitamins, choline, and carotenoids (4). Yolk sphingolipids further contribute to membrane organization and extracellular matrix regulation, underscoring the egg’s intrinsic physiological functionality. Owing to their capacity to carry both lipophilic and hydrophilic compounds, eggs are increasingly recognized as effective vehicles for dietary bioactives (5). Advances in nutritional science, combined with feed-based modulation strategies, have enabled the development of functional eggs, such as omega-3 (ω-3) enriched eggs, which offer a practical approach to improving population-level nutrient intake through habitual food consumption (6).

The growing emphasis on diet-based disease prevention has gained growing interest in nutrient-enriched eggs as functional foods that deliver health-promoting compounds beyond their natural high-quality protein (7). The lipid-rich yolk provides an efficient matrix for incorporating fat-soluble bioactives, enabling targeted enrichment of omega-3 polyunsaturated fatty acids (ω-3 PUFAs), carotenoids (lutein and zeaxanthin), vitamin E, lycopene, and essential trace minerals such as selenium and iodine (8, 9). Among these, ω-3 PUFAs are the most extensively studied; dietary supplementation with fish oil, flaxseed, or algae increases yolk eicosatetraenoic acid (EPA) and docosahexaenoic acid (DHA), supporting cardiovascular and cognitive health. However, higher ω-3 levels increase lipid oxidation risk, making concurrent enrichment with antioxidants essential to stabilize yolk lipids and enhance systemic oxidative defense (10). Despite advances, research remains fragmented across nutrient types, feeding strategies, and biological outcomes, limiting integrated understanding of nutrient deposition mechanisms, oxidative stability, and health efficacy. This review therefore summarizes strategies for ω-3 PUFA, antioxidant, and trace mineral enrichment, integrating metabolic transport from intestinal absorption to hepatic processing, lipoprotein assembly, and yolk deposition, to provide mechanistic insights and guide future functional egg development. By comparing deposition efficiency, functional outcomes, and identifying knowledge gaps, we provide insights to guide future precision nutrition strategies and industrial applications of functional eggs. Unlike previous reviews that primarily focus on single nutrient categories or enrichment outcomes, this work offers a mechanistic and strategy-oriented perspective to advance the development of next-generation functional eggs.

## 2 Eggs as nutrient delivery systems

### 2.1 Biological basis and bioactive components

Structurally, a chicken egg comprises the shell with membranes, albumen, and yolk, which account for approximately 9.5, 63, and 27.5% of the whole egg, respectively (11). The edible portion primarily consists of water (~74%), protein (~12%), lipid (~12%), carbohydrates (<1%), and ash (~1%), with proteins distributed across both albumen and yolk, while lipids, fat-soluble vitamins, and most minerals are concentrated in the yolk (12). Egg proteins, including ovalbumin, ovotransferrin, lysozyme, phosvitin, and other yolk-specific proteins such as lipoproteins, provide high-quality, digestible amino acids and exhibit multiple biological functions, such as antioxidant, antimicrobial, and anti-inflammatory effects (4, 13–15). Proteomic analyses have identified nearly a thousand distinct egg proteins, highlighting their functional and therapeutic potential for health maintenance and disease management (14). Egg yolk lipids are primarily triacylglycerols (~65%), phospholipids (~30%), and cholesterol (~4%), with phospholipids such as phosphatidylcholine and phosphatidylethanolamine constituting 28–30% of total yolk lipids (16–18). These phospholipids are highly bioavailable (>90%), efficiently incorporated into plasma high-density lipoproteins, and play key roles in lipid metabolism regulation. The fatty acid composition of yolk lipids can be modulated via maternal diet to enrich polyunsaturated fatty acids (PUFAs), including ω-3 s α-linolenic acid (ALA), EPA, and DHA, thereby improving the ω-6: ω-3 ratio, which is critical for cardiovascular and cognitive health (19, 20). Eggs are also a natural source of bioactive carotenoids, primarily lutein and zeaxanthin, which exhibit higher bioavailability than plant-derived forms (21, 22). These carotenoids preferentially accumulate in the retina, enhance macular pigment density, and protect against age-related macular degeneration (23). Furthermore, eggs provide fat-soluble vitamins (A, D, E, K), choline, and trace minerals such as selenium and iodine, contributing to antioxidant defense, immune modulation, neurodevelopment, and other physiological functions (6, 24). Collectively, eggs provide a compact, nutrient-dense matrix of high-quality proteins, lipids, vitamins, minerals, and bioactive compounds. Their structural and biochemical characteristics, combined with the ability to modulate nutrient composition through feed enrichment, make eggs an ideal natural delivery system for functional nutrients and bioactive compounds.

### 2.2 Absorption and bioavailability

The absorption and bioavailability of egg-derived nutrients are enhanced by the egg’s unique biochemical matrix. Lipophilic compounds, including phospholipids, carotenoids, fat-soluble vitamins, and ω-3 PUFAs, are efficiently solubilized within yolk lipids, improving intestinal uptake and systemic availability (25, 26). For example, phosphatidylcholine is preferentially incorporated into high-density lipoprotein (HDL) particles, promoting favorable lipid profiles and supporting cardiovascular health (27).

Egg proteins are highly digestible, with cooked egg white achieving ~91% digestibility compared to ~51% for raw egg, providing rich essential amino acids that support muscle protein synthesis (28, 29). Specific bioactive proteins, such as lysozyme and ovotransferrin, can partially resist gastrointestinal degradation, allowing them to exert systemic antioxidant and antimicrobial effects (30, 31). The bioavailability of lutein and zeaxanthin is influenced by cooking methods, with boiling optimizing absorption (32, 33), and their incorporation into micelles is further enhanced by yolk phospholipids (34). Likewise, maternal dietary enrichment of eggs with ω-3 PUFAs, vitamins, or trace elements ensures that these nutrients are deposited in forms that are efficiently absorbed, maximizing the functional benefits of enriched eggs (35, 36). In summary, the unique yolk matrix and protein composition of eggs enhance the bioavailability of lipophilic and hydrophilic nutrients, ensuring that proteins, fatty acids, vitamins, and carotenoids are efficiently absorbed and functionally active.

### 2.3 Comparison with other food carriers

**Figure 1:**
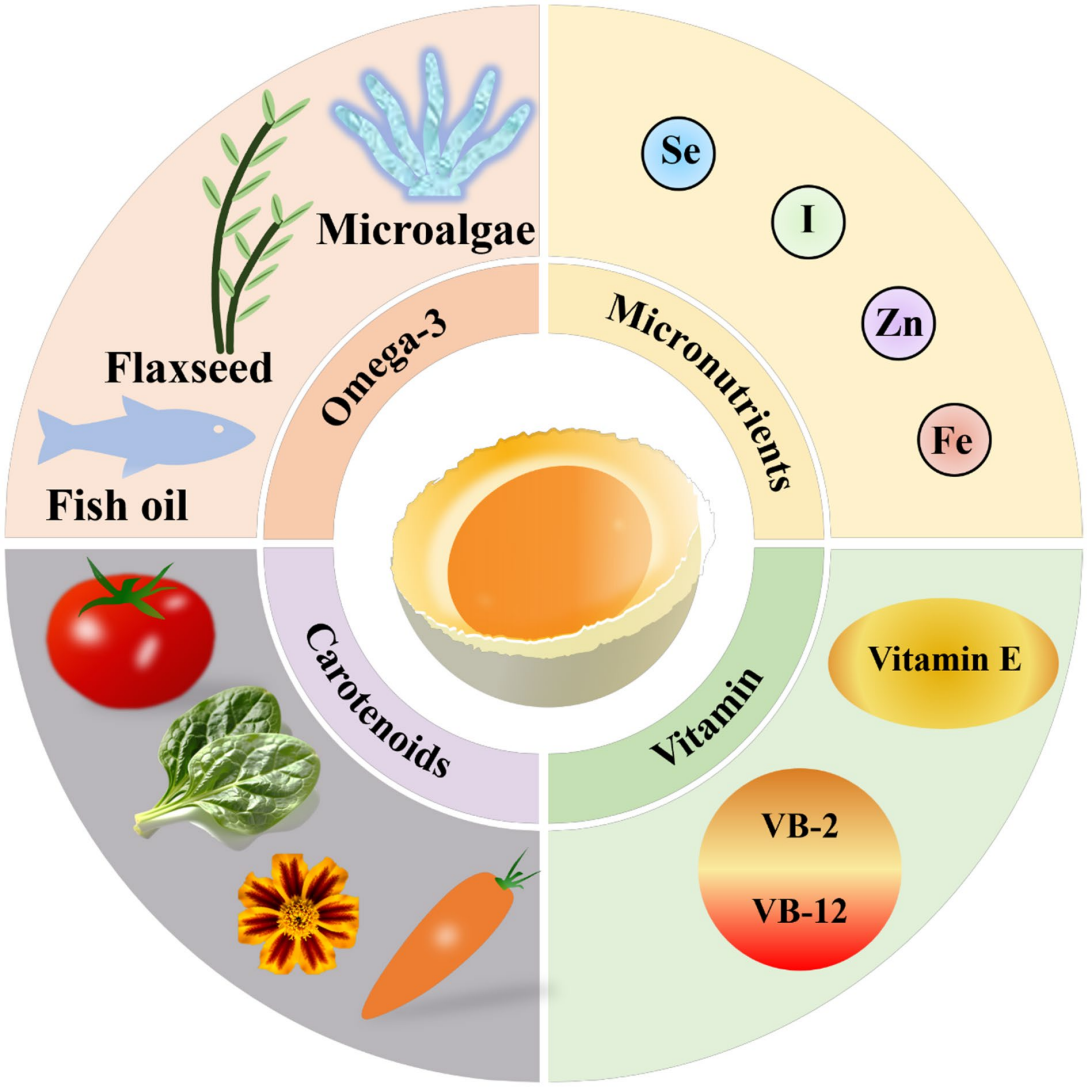
Schematic overview of egg-based nutrient fortification strategies and representative bioactive components. Major approaches for functional egg production are illustrated, highlighting ω-3 PUFAs, antioxidants (e.g., carotenoids, vitamin E), vitamins (VB₂, VB₁₂), and trace elements (selenium, iodine, zinc, iron). ω-3 PUFAs are enriched via dietary supplementation (fish oil, flaxseed, microalgae), while antioxidants, vitamins, and minerals are incorporated through targeted feed modulation. Nutrients are absorbed in the intestine, metabolized in the liver, and transported to the ovary for deposition into the yolk. This schematic emphasizes the integrated nutrient matrix of enriched eggs and their potential as efficient biological carriers for functional food development.

Eggs represent a uniquely efficient vehicle for nutrient delivery compared with other common dietary carriers such as dairy products, dietary supplements, or functional beverages. Their compact matrix naturally combines high-quality proteins, lipids, fat- and water-soluble vitamins, minerals, and bioactive compounds in a balanced form that supports both stability and bioavailability. Lipophilic nutrients, including carotenoids, fat-soluble vitamins, phospholipids, and ω-3 PUFAs, are solubilized within yolk lipids, enhancing intestinal absorption and systemic distribution, often exceeding the bioefficacy of isolated supplements (5, 37). Eggs also allow co-delivery of enriched nutrients with endogenous components such as phospholipids, choline, and antioxidant peptides, enabling synergistic physiological effects that are difficult to achieve with pill-based formulations. Moreover, egg proteins are highly digestible and provide bioactive peptides that can exert systemic antioxidant, immunomodulatory, or metabolic effects. As a widely consumed staple food, eggs combine high consumer acceptance, culinary versatility, and sustained dietary compliance, strengthening their translational potential as functional foods. Collectively, these characteristics position eggs as a superior and practical platform for functional fortification, capable of delivering multiple nutrients simultaneously while maintaining stability, bioavailability, and physiological efficacy, in contrast to many conventional supplements or fortified products. This capacity underpins their central role in developing functional eggs enriched with ω-3 PUFAs, antioxidants, and essential minerals, as discussed in subsequent sections (Figure 1).

## 3 ω-3 enrichment in eggs

### 3.1 Physiological roles of ω-3 PUFAs

ω-3 PUFAs, primarily ALA, EPA, and DHA, play essential roles in cardiovascular, metabolic, neurological, and immune health (38). ALA is an essential fatty acid that must be supplied by the diet, whereas EPA and DHA are directly incorporated into cellular membranes, supporting membrane fluidity, eicosanoid balance, and neurocognitive function (19). Extensive evidence showed that EPA and DHA provide cardiometabolic protection by lowering circulating triglycerides, improving endothelial function, enhancing vascular compliance, and reducing arrhythmic events (39). The involvement in reverse cholesterol transport, modulation of LDL particle size, and maintenance of arterial elasticity collectively contributes to a reduced risk of atherosclerosis and insulin resistance. In metabolic syndrome, ω-3 PUFAs have demonstrated benefits in attenuating hyperlipidemia, hypertension, hyperglycemia, hepatic steatosis, and endothelial dysfunction (39, 40). In the central nervous system, DHA is a key structural component of neuronal membranes and supports synaptic plasticity, cognitive performance, and early-life neurodevelopment (41). Additionally, ω-3 PUFAs promote the resolution of inflammation through the formation of specialized pro-resolving mediators, including resolvins and protectins, thereby exerting systemic anti-inflammatory and immunomodulatory effects (42).

Eggs naturally supply high-quality protein, phospholipids, fat-soluble vitamins (A, D, E, K), B vitamins, minerals, and other bioactive lipids (13). Enriching eggs with ω-3 PUFAs offers an accessible and cost-effective dietary strategy to improve population-level intake. Flaxseed supplementation in layers is widely employed to increase yolk ALA and DHA content, producing ω-3-enriched eggs with up to five times the ω-3 PUFAs content of conventional eggs (39). Consuming these enriched eggs has been shown to reduce serum cholesterol and triglyceride levels and to shift LDL particles toward less atherogenic forms, thereby improving cardiometabolic risk profiles (43).

### 3.2 Dietary strategies for ω-3 egg enrichment

Eggs respond rapidly and predictably to dietary manipulation of their fatty acid composition, making laying hens an efficient biological system for enriching ω-3 PUFAs, including ALA, EPA, and DHA. Because birds lack Δ12- and Δ15-desaturases, both n-6 and ω-3 PUFAs must be supplied through the diet, and the endogenous conversion of ALA to EPA/DHA is inherently limited, with desaturation at the Δ6 position representing a major metabolic bottleneck (44). Consequently, efficient yolk enrichment depends on dietary sources that provide preformed long-chain ω-3 PUFAs or highly bioavailable precursors.

Fish oil is the most direct source of preformed EPA and DHA and consistently elevates yolk ω-3 PUFAs. For example, inclusion of 1.5% fish oil increased total ω-3 PUFAs from 205 to 327 mg/100 g egg (45). However, higher inclusion levels can introduce undesirable fishy flavors and increase lipid oxidation susceptibility, requiring careful formulation and antioxidant support (46). Microalgae and microalgae-derived oil offer a sustainable, high-purity source of DHA while avoiding the sensory issues associated with marine oils (47). Comparative studies show that algal oil can achieve DHA deposition comparable to or exceeding that of fish oil, with improved flavor stability, making algal DHA an increasingly attractive option (48). Flaxseed or linseed oil, rich in ALA, effectively raises yolk ALA concentrations. Reported increases range from 1.5% to nearly 10% of total fatty acids when 1–4% flaxseed oil is included in the diet. Although hens convert ALA to EPA and DHA only inefficiently, flaxseed remains a widely used, cost-effective plant source for increasing total ω-3 PUFAs (49). Other emerging sources, including chia, perilla, and camelina seeds, as well as encapsulated ω-3 lipids, provide additional dietary ALA or stabilized EPA/DHA forms while enhancing oxidative stability during storage and cooking (47). Overall, evidence from multiple feeding trials demonstrates that dietary inclusion of these oil sources can increase yolk ALA, EPA, and DHA by 2- to 10-fold while substantially reducing the ω-6/ω-3 ratio. Comparatively, fish oil achieves the highest DHA/EPA deposition but may negatively impact flavor and oxidative stability; algal oil offers sustainable DHA enrichment with improved sensory quality; and flaxseed provides a cost-effective, plant-based source of ALA, albeit with limited conversion to long-chain ω-3 PUFAs. Therefore, the choice of feed source must balance enrichment efficiency, sensory quality, oxidative stability, and production performance (47).

### 3.3 Mechanisms of fatty acids enrichment in eggs

**Figure 2:**
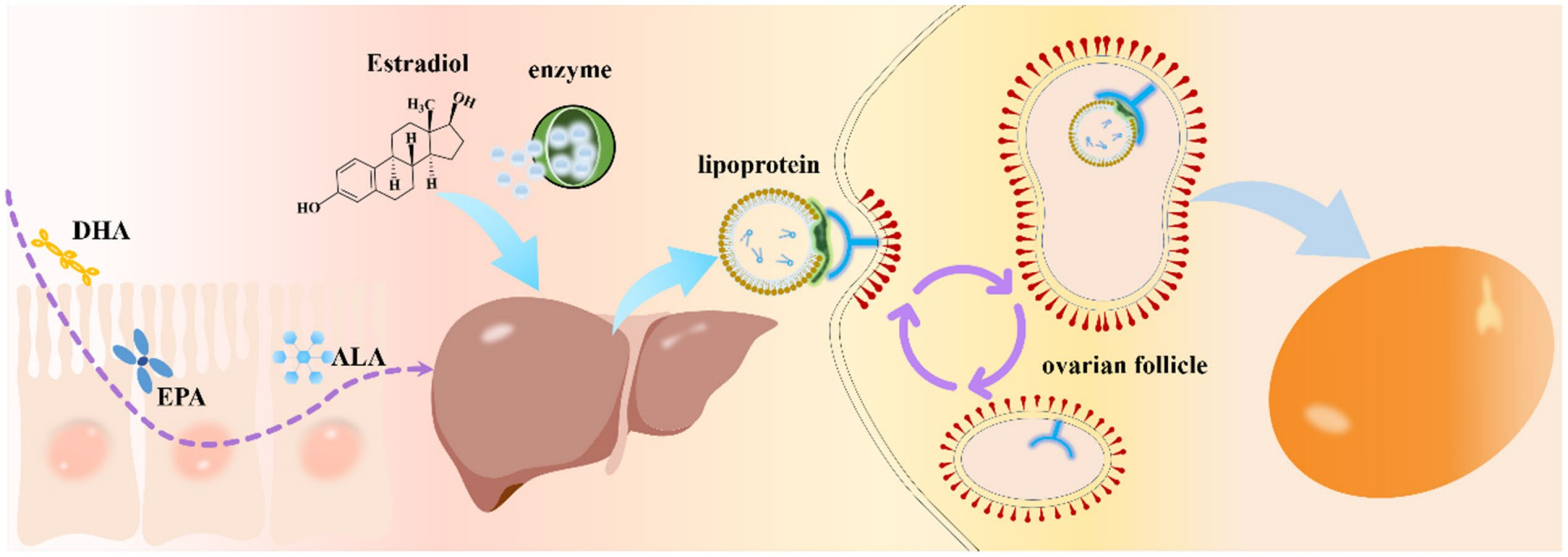
Proposed regulatory route for ω-3 PUFAs deposition in eggs. Dietary ω-3 PUFAs (ALA, EPA, and DHA) are absorbed by intestinal epithelial cells and transported to the liver via portal circulation. In the liver, they undergo metabolic conversion, including elongation and desaturation, and are incorporated into triglycerides and phospholipids. These lipids are assembled into very-low-density lipoproteins (VLDL) and secreted into the systemic circulation. Circulating VLDL delivers ω-3 PUFAs to the ovary, where receptor-mediated uptake by ovarian follicles transfers them into developing oocytes. The deposited ω-3 PUFAs enrich the egg yolk lipid fraction, with DHA accumulation being a key outcome. This intestine-liver-ovary axis highlights hepatic lipid metabolism and lipoprotein transport as central regulatory steps in feed-based ω-3 PUFAs enrichment.

Dietary fatty acids, especially ω-3 PUFAs such as EPA and DHA, as well as their precursor ALA, must undergo a series of coordinated digestive, metabolic, and selective deposition processes before being incorporated into the developing yolk (50). Following ingestion, these lipids are released and absorbed in the gastrointestinal tract, transported to the liver for metabolic routing and limited bioconversion, assembled into yolk-targeted lipoproteins, and ultimately taken up by ovarian follicles via highly selective receptor-mediated pathways. A comprehensive understanding of each step along this metabolic axis is critical for optimizing nutritional strategies aimed at producing ω-3 PUFA-enriched eggs. Overall, the process can be conceptualized in three interrelated stages, as illustrated in Figure 2: (1) intestinal digestion, absorption, and transport, (2) hepatic uptake, enzymatic conversion, and lipoprotein assembly, and (3) ovarian follicle uptake and selective deposition into the yolk.

#### 3.3.1 Intestinal absorption and transport

Upon ingestion, dietary triglycerides (TG) enriched with ω-3 PUFAs, including ALA, EPA, and DHA, undergo coordinated emulsification, enzymatic hydrolysis, and transporter-mediated uptake in the avian gastrointestinal tract (51, 52). Bile salts/acids disperse lipid droplets into smaller micellar structures, allowing pancreatic lipase to selectively hydrolyze TG at the sn-1 and sn-3 positions, generating free fatty acids (FFAs) and 2-monoacylglycerols (2-MAGs) (53). Importantly, the positional distribution of ω-3 PUFAs within TG molecules critically influences their digestive fate. PUFAs esterified at the sn-2 position are retained in 2-MAGs, which are more efficiently absorbed by enterocytes than free fatty acids (54). This structural preference provides a mechanistic basis for improved intestinal bioavailability of DHA-rich lipids. Mixed micelles composed of FFAs, 2-monoacylglycerols, bile salts, and phospholipids diffuse across the unstirred water layer to the brush-border membrane (BBM) (55). At the BBM, the acidic microenvironment promotes micelle dissociation and protonation of FFAs. These processes facilitate fatty acid uptake through both passive diffusion and transporter-mediated pathways (55). Although avian lipid absorption has been less extensively characterized than in mammals, accumulating evidence indicates conservation of key fatty acid transport systems. CD36/fatty acid translocase (FAT), highly expressed in the proximal intestine, mediates long-chain fatty acid uptake and is dynamically regulated by post-translational modifications such as palmitoylation, thereby influencing lipid flux toward portomicron assembly (56–58). Fatty acid transport protein 4 (FATP4) further promotes intracellular retention of ALA through acyl-CoA formation, while members of the fatty acid-binding protein (FABP) family (I-FABP, L-FABP, FABPpm) chaperone ω-3 PUFAs toward the endoplasmic reticulum for re-esterification (58–60). Within enterocytes, absorbed lipids are re-esterified into TG and phospholipids and packaged into portomicrons, the avian equivalent of chylomicrons. Unlike mammals, portomicrons enter the portal vein rather than the lymphatic system, delivering dietary lipids directly to the liver and establishing the liver as the primary metabolic gateway for subsequent yolk lipid deposition.

#### 3.3.2 Hepatic uptake and conversion

In laying hens, hepatic clearance of circulating portomicron triglycerides occurs via lipoprotein lipase-mediated hydrolysis and receptor-mediated endocytosis, releasing FFAs for intracellular metabolism (61). Within hepatocytes, ω-3 PUFAs occupy a metabolic decision point, where they may be directed toward β-oxidation for energy production or retained for structural incorporation and further elongation and desaturation (62). The endogenous conversion of ALA (18:3n-3) to long-chain ω-3 PUFAs proceeds via a conserved enzymatic cascade involving Δ6-desaturase, elongases (ELOVL2/5), and Δ5-desaturase, with DHA synthesis requiring an additional peroxisomal β-oxidation step of C24 intermediates (63, 64). However, the inherently limited activity of these enzymes in poultry constrains conversion efficiency, explaining why dietary DHA generally produces more robust yolk enrichment than precursor ALA (65). Beyond biosynthesis, the liver plays a decisive role in selectively retaining ω-3 PUFAs for yolk deposition. DHA is preferentially esterified into phosphatidylcholine (PC) and triacylglycerol pools, often occupying the sn-2 position of phospholipids, which enhances molecular stability and reduces susceptibility to oxidative catabolism (65). Estrogen further amplifies hepatic lipogenic capacity and induces the synthesis of yolk precursor lipoproteins, including yolk-targeted very-low-density lipoprotein (VLDLy) and vitellogenin (VTG), thereby synchronizing ω-3 PUFA metabolism with the reproductive cycle. Collectively, these processes position the liver as a metabolic hub that integrates dietary supply, enzymatic capacity, hormonal regulation, and selective lipid packaging to determine the efficiency of ω-3 PUFA transfer to the ovary.

#### 3.3.3 Lipoprotein assembly and follicular uptake

Hepatic assembly of yolk precursor particles yields two functionally specialized carriers: VLDLy and VTG. VLDLy is a small, estrogen-modified VLDL subclass (≤40 nm) characterized by resistance to peripheral lipoprotein lipase hydrolysis, allowing it to bypass extrahepatic tissues and deliver DHA-rich TG and phospholipids directly to the ovary (65, 66). VTG, a high-molecular-weight phospholipoglycoprotein, provides DHA-enriched PC and PE required for yolk membrane formation and structural organization. Growing ovarian follicles express high levels of the very-low-density lipoprotein receptor (VLDLR), enabling clathrin-dependent endocytosis of circulating VLDLy and VTG. Following internalization, these particles undergo minimal biochemical remodeling and are efficiently incorporated into yolk spheres (66). Therefore, yolk fatty acid composition closely mirrors the lipid profile of hepatic VLDLy and VTG, providing a mechanistic explanation for the rapid and dose-responsive modulation of yolk ω-3 PUFAs levels following dietary intervention.

#### 3.3.4 Selective deposition of DHA in yolk

Among dietary fatty acids, DHA exhibits a pronounced selective advantage for yolk deposition. This preference arises from coordinated mechanisms across the intestinal-hepatic-ovarian axis. DHA displays reduced susceptibility to mitochondrial β-oxidation, stronger affinity for phospholipid esterification, and preferential incorporation into PC, enhancing its metabolic retention in the liver. DHA-enriched PC is efficiently packaged into VLDLy and demonstrates increased oxidative stability during systemic transport, facilitating intact delivery to developing follicles (67, 68). At the ovarian level, receptor-mediated uptake and intracellular handling further favor DHA incorporation into the granular yolk layer, where it accumulates predominantly within phospholipid fractions. Consequently, yolk DHA content increases in a dose-dependent manner with dietary supplementation until physiological saturation is reached (68). Together, these multi-level selection mechanisms ensure that DHA is disproportionately enriched in egg yolk relative to other fatty acids, highlighting the intestinal-hepatic-ovarian axis as an integrated and targetable system for precision ω-3 PUFAs fortification.

## 4 Antioxidant enrichment in eggs

### 4.1 Major antioxidants and their physiological functions

Antioxidant-enriched eggs represent an important category of functional eggs aimed at improving both egg quality and human health through targeted enhancement of yolk antioxidant capacity. Approximately 36% of the edible portion of an egg consists of lipids, nearly all of which are concentrated in the yolk and organized into lipoprotein structures rich in triacylglycerols, phospholipids, and cholesterol (69, 70). Although carotenoids account for less than 1% of total yolk lipids, they are the primary determinants of yolk pigmentation and contribute substantially to the antioxidant and anti-inflammatory properties of eggs (69). Dietary supplementation of laying hens with vitamin E, carotenoids, and plant-derived polyphenols is widely employed to enhance yolk antioxidant content. This targeted enrichment improves oxidative stability, enhances nutritional value, and confers physiologically relevant health benefits, thereby positioning antioxidant-enriched eggs as a promising strategy for the development of value-added functional foods.

#### 4.1.1 Carotenoids: lutein, zeaxanthin, and β-carotene

Carotenoids are lipophilic isoprenoid molecules synthesized primarily by photosynthetic organisms. More than 600 carotenoids have been identified, with around 50 presents in the human diet and 14 detectable in human plasma (71). Because humans cannot synthesize carotenoids, dietary intake is essential. Egg yolk is a unique and highly bioavailable source of carotenoids such as lutein, zeaxanthin, β-cryptoxanthin, and β-carotene (72, 73). The lipid-rich yolk matrix, particularly its triacylglycerol and phospholipid micelles, eastly enhances carotenoid micellization and intestinal absorption compared with plant sources (74, 75). Consequently, carotenoid-enriched eggs not only elevate dietary carotenoid intake but also significantly improve the bioavailability of carotenoids from co-consumed foods, such as salads (74). Lutein and zeaxanthin are major components of the macular pigment and exert visual-protective effects through several mechanisms (76–78), including scavenging singlet oxygen and reactive oxygen species (ROS), reducing oxidative damage to lipids, proteins, and DNA, and supporting glutathione homeostasis under oxidative stress. Accumulating evidence further indicates that carotenoid supplementation reduces lipid peroxidation, attenuates oxidized LDL levels, and protects against early atherosclerotic changes (79–81). Given these functions, carotenoid-enriched eggs serve as an effective strategy to enhance dietary intake of bioactive pigments and support ocular and cardiovascular health.

#### 4.1.2 Vitamin E (α-tocopherol)

Vitamin E is the primary lipid-soluble, chain-breaking antioxidant in biological membranes, where inhibits PUFAs peroxidation and preserves cell membrane integrity (82). Eggs naturally provide approximately 1.1 mg of vitamin E, corresponding to ~8.5% of the recommended daily intake (15). Supplementing hen diets with α-tocopherol can increase yolk vitamin E content up to 150% of the recommended daily allowance without affecting sensory quality (83). Beyond supporting immune function, vitamin E protects LDL and HDL particles from oxidative modification and is associated with reduced cardiovascular disease risk (84). In eggs, elevated vitamin E levels improve yolk oxidative stability, reducing PUFAs degradation during storage and cooking.

#### 4.1.3 Polyphenols and plant-derived antioxidants

Polyphenols, flavonoids, and other plant-derived compounds exhibit anti-inflammatory, anti-glycation, ROS-scavenging, and metal-chelating properties (85). Although chicken cannot synthesize polyphenols, dietary inclusion of polyphenol-rich feed ingredients (e.g., green tea extract, grape seed extract, herbal extracts) can enhance their deposition into egg yolk or albumen (86). These compounds represent an emerging frontier in egg enrichment strategies, with the potential to act synergistically with lipid-soluble antioxidants such as vitamin E and carotenoids. Through such interactions, plant-derived polyphenols may further enhance oxidative stability and augment the functional properties of antioxidant-enriched eggs.

#### 4.1.4 Antioxidant minerals: selenium and iodine

Selenium contributes to antioxidant capacity through its role in selenoproteins such as glutathione peroxidase (GPx) and thioredoxin reductase (TrxR) (87, 88). Consumption of selenium-enriched eggs has been shown to enhance antioxidant enzyme activity in humans, highlighting their potential to improve systemic oxidative status (89, 90). Iodine also exhibits indirect antioxidant effects by maintaining thyroid hormone homeostasis. Deficiency-induced TSH overstimulation can lead to increased hydrogen peroxide production (91). Accordingly, iodine-enriched eggs are of interest not only for ensuring adequate iodine intake but also for supporting oxidative balance and endocrine health.

### 4.2 Strategies for antioxidant enrichment

Eggs, with their high lipid content, provide an ideal matrix for lipophilic antioxidants, including carotenoids (lutein, zeaxanthin), vitamin E, selenium, iodine, lycopene, and ω-3 PUFAs (92). Dietary supplementation of laying hens with ω-3-ich ingredients such as fish oil, flaxseed, microalgae effectively increase yolk ω-3 PUFAs content. However, elevated levels of PUFAs also increase susceptibility to lipid peroxidation, highlighting the importance of co-supplementation with antioxidants, such as vitamin E and carotenoids, to enhance both oxidative stability and the nutritional benefits of enriched eggs (10).

Carotenoids, the main lipophilic pigments in yolks, are highly dependent on the hen’s diet. Natural sources such as marigold (*Tagetes erecta*) and alfalfa (*Medicago sativa*) provide lutein, while maize (*Zea mays*) and red peppers (*Capsicum annuum*) supply zeaxanthin and capsanthin. Chemically synthesized carotenoids, including canthaxanthin, β-apo-8′-carotene, and β-apo-8′-carotenoic acid esters, are also used to modulate yolk pigmentation and antioxidant capacity (72, 93). Lutein and zeaxanthin play key roles in mitigating oxidative stress and supporting retinal and skin health. Biofortified eggs can achieve up to a 15-fold increase in yolk lutein (~1.9 mg per egg) with higher bioavailability than dietary supplements or plant sources such as spinach (83, 94). Natural carotenoids are derived from microalgae (*Spirulina, Chlorella*), yeast (*Phaffia rhodozyma, Rhodotorula rubra*), bacteria (*Paracoccus* spp.), plants (marigold, basil, Stevia, and spinach), plant by-products (tomato pomace, carrot derivatives, and grape seed powder), and animal by-products (crab meal, golden snail egg powder) (95–100). Additionally, biofortified crops, such as lutein- or zeaxanthin-enriched maize, are also employed to enhance yolk carotenoid content (101). While synthetic carotenoids remain common in conventional feed, consumer preference for natural sources has increased, particularly in organic egg production (102).

Other antioxidants, including vitamin E, folate, selenium, and iodine, can also be enriched through maternal diet modification. Vitamin E protects membrane lipids from peroxidation and can reach ~20 mg per egg, exceeding daily human requirements while preserving unsaturated fatty acids (103). Folate, particularly in the form of 5-methyltetrahydrofolate (5-MTHF), is a potent antioxidant that remains stable during cooking, and can supply up to 12.5% of the recommended daily intake per egg (104, 105). Selenium and iodine are efficiently deposited in yolks, potentially providing 50% of daily human requirements (106). In summary, antioxidant enrichment of eggs relies on natural feed ingredients, synthetic compounds, and maternal diet modification to achieve targeted deposition of bioactive compounds in yolk lipids or phospholipid fractions. These strategies improve oxidative stability and enhance the nutritional and functional properties of eggs for human consumption.

### 4.3 Mechanisms of carotenoid enrichment in eggs

Dietary carotenoids are absorbed and transported to egg yolks through a multistage physiological process (107). In the duodenum, digestive enzymes release carotenoids from the food matrix, which are then solubilized in mixed micelles composed of fatty acids, phospholipids, cholesterol, bile salts, and monoacylglycerols to facilitate intestinal absorption (108, 109). Carotenoids enter enterocytes via passive diffusion across the unstirred water layer and active transport mediated by scavenger receptor class B type 1 and cluster of differentiation 36 (110, 111). Certain carotenoids, such as lutein esters, are hydrolyzed by intestinal lipases to free forms for more efficient uptake (112). Within enterocytes, carotenoids may be enzymatically cleaved by β-carotene-15,15′-oxygenase or β-carotene-9′,10′-oxygenase to generate bioactive metabolites. While unprocessed carotenoids are incorporated into chylomicrons along with lipids and subsequently released into the lymphatic system, ultimately reaching the circulation (108, 111, 113, 114).

In the liver, carotenoids are incorporated into lipoproteins, VLDL, LDL, and HDL, for transport to peripheral tissues and the ovary (115–117). Polar carotenoids like lutein and zeaxanthin distribute more evenly between LDL and HDL, whereas nonpolar carotenoids, including β-carotene, are mainly carried by VLDL and LDL (109, 118). The ovary selectively uptakes VLDL particles carrying VLDLy (~30 nm), which are resistant to lipoprotein lipase-mediated hydrolysis and recognized by LR8 receptors on developing oocytes, enabling targeted carotenoid deposition into the yolk (115, 119, 120). Carotenoid deposition occurs rapidly, yet yolk pigmentation stabilizes over 6–14 days depending on yolk size and oocyte growth stage (121). Studies indicate that yolk color reaches a plateau after 7–12 days of supplementation, independent of dosage, reflecting the physiological kinetics of carotenoid accumulation (122–125). Overall, carotenoid deposition in eggs involves coordinated release from the diet, micellar solubilization, receptor-mediated intestinal uptake, lipoprotein-mediated hepatic transport, and selective ovarian incorporation. Understanding these mechanisms is essential for optimizing dietary strategies to produce antioxidant-enriched eggs with enhanced carotenoid content and improved nutritional and functional properties (107, 126, 127).

## 5 Micronutrients enriched-eggs

### 5.1 Major micronutrients and their physiological roles

Global production approaching 80 million tons and an average per capita consumption of approximately 161 eggs per year. Their broad accessibility and habitual consumption make eggs a practical dietary carrier for micronutrient delivery (128). Experimental and observational studies indicate that eggs can efficiently accumulate and maternally transfer essential trace elements, including selenium (Se), iodine (I), iron (Fe), and zinc (Zn), into the yolk (129). Although the physicochemical characteristics of the yolk lipid-protein matrix are often cited as supporting high micronutrient bioavailability, quantitative comparisons with other fortified foods or supplements remain limited.

The concept of micronutrient-enriched eggs has evolved alongside the recognition of eggs as functional foods capable of addressing dietary inadequacies. In low- and middle-income regions, where micronutrient deficiencies are highly prevalent, eggs have been promoted as a cost-effective and culturally acceptable intervention (130). Several randomized and quasi-experimental studies have reported improvements in growth indicators and overall nutritional status in infants and young children following regular egg consumption, supporting the physiological relevance of egg-derived micronutrients during early development (131–133). However, these benefits appear context-dependent, varying with geographic region, baseline dietary patterns, and coexisting nutrient deficiencies. Evidence from high-income countries further indicates that insufficient intake of certain micronutrients, including vitamin E, iron, and zinc, persists despite generally adequate food availability, suggesting that egg-based strategies may also have relevance beyond undernourished populations (133, 134). However, long-term population-level evidence linking egg-derived micronutrient intake to clinically meaningful health outcomes remain limited.

Among the trace elements targeted for egg fortification, selenium and zinc have received particular attention due to their fundamental roles in antioxidant defense, immune regulation, thyroid hormone metabolism, and growth (135). Selenium-enriched eggs predominantly provide selenium in organic forms, which exhibit superior absorption and biological efficacy compared with inorganic selenium sources (136). Similarly, zinc-enriched eggs contribute to immune competence, enzymatic function, and early growth and neurodevelopment (137). In addition, iron- and iodine-enriched eggs have been explored as nutritional strategies to prevent iron-deficiency anemia and iodine deficiency disorders, respectively, with particular relevance for vulnerable groups such as infants, pregnant women, and adults.

### 5.2 Strategies for micronutrient enrichment

Eggs are naturally rich in essential nutrients, and their mineral composition can be substantially enhanced through targeted dietary interventions and advanced nutritional technologies. Current poultry nutrition strategies aim to increase the concentrations of minerals, such as chromium (Cr), iron (Fe), zinc (Zn), selenium (Se), iodine (I), and manganese (Mn), in eggs through feed-based interventions, thereby improving their nutritional value. The efficiency of mineral deposition into eggs is determined by multiple interacting factors, including the chemical form of the mineral (organic vs. inorganic), feed formulation, hen physiology, mineral-nutrient interactions, and production systems (e.g., conventional, free-range, or organic housing) (138, 139). Compared with conventional fortified foods or dietary supplements, mineral-enriched eggs offer several advantages: they are affordable, widely consumed, culturally accepted, and provide minerals within a natural, highly bioavailable biological matrix (140). Consequently, micronutrient-enriched eggs have attracted increasing attention as a scalable and food-based strategy to address “hidden hunger,” particularly in low-income populations, while remaining relevant in high-income countries where suboptimal micronutrient intake persists.

Among available approaches, the enrichment and biological conversion of selenium and zinc into organic forms via plant- or animal-derived feed ingredients has emerged as an effective strategy to enhance mineral efficacy (141). The egg matrix itself further facilitates organic mineral transformation and stabilization, which may improve intestinal absorption and metabolic utilization in humans. Despite progress in enhancing mineral deposition, the nutritional functionality and biological activity of selenium- and zinc-enriched eggs, as well as mineral-enriched eggs more broadly, remain insufficiently evaluated, with most studies focusing on compositional outcomes rather than long-term health effects (129, 142). This synthesizes current feed-based fortification approaches, including the use of inorganic salts, organic chelates, mineral-enriched plants and yeasts, and emerging nano- and biologically transformed mineral sources. In addition, key determinants of mineral deposition efficiency, such as mineral chemical form, dosage, interactions with other nutrients, hen physiological status, and production system, are critically considered.

### 5.3 Mechanisms of mineral enrichment in eggs

The deposition and enrichment of minerals in eggs is a tightly regulated, compartment-specific process that integrates dietary mineral supply, hen physiology, and embryonic developmental demands. Following ingestion, minerals are absorbed in the gastrointestinal tract of laying hens, with absorption efficiency strongly influenced by mineral chemical form (organic vs. inorganic), interactions with other dietary components, and the presence of anti-nutritional factors such as phytates (143). Organic mineral sources, including selenium-methionine, zinc-amino acid chelates, and mineral-enriched yeasts, generally exhibit superior intestinal bioavailability compared with inorganic salts, thereby facilitating more efficient systemic transport and subsequent deposition into eggs (144). After absorption, minerals are transported via the bloodstream and selectively deposited into different egg compartments through two anatomically and functionally distinct maternal pathways: ovarian transfer into the yolk and oviductal secretion into the albumen, shell membranes, and eggshell (145). This dual-pathway system underlies the pronounced mineral heterogeneity observed between yolk and albumen. For example, phosphorus (P), Zn, Cu, Mn, and Fe are predominantly localized in the yolk, whereas sodium (Na) and potassium (K) are mainly associated with the albumen. Quantitative analyses of fresh eggs from 50-week-old Cobb 500 breeder hens indicate that the yolk accounts for over 94% of total Mn and P, while contributing less than 20% of total Na and approximately one-third of total K, highlighting the yolk’s primary function as a storage reservoir for trace minerals essential to embryonic development (146).

The accumulation of minerals in the yolk reflects its unique lipid- and protein-rich matrix, which enables the stabilization and long-term retention of protein-bound and lipophilic mineral complexes (147, 148). Albumen primarily serves as a medium for water-soluble and protein-associated electrolytes that support early embryonic homeostasis (149). Importantly, once minerals are deposited into the yolk, their concentrations remain relatively stable and are respond only weakly to short-term dietary changes, indicating that egg mineral composition is governed by stringent physiological control rather than simple proportional transfer from the maternal diet (144). Mineral deposition efficiency is further modulated by hen-specific and production-related factors, including age, laying stage, endocrine regulation, stress status, and housing system (144). As maternal deposition constitutes the sole source of minerals available to the developing embryo during incubation, both the quantity and distribution pattern of minerals within the egg are critical determinants of embryonic viability, skeletal development, immune competence, and post-hatch performance. Collectively, effective egg mineral enrichment depends on the interplay of dietary fortification, maternal absorption and transport, and compartment-specific deposition. Understanding these coordinated mechanisms is essential for designing strategies to optimize egg mineral content and support both embryonic development and post-hatch performance.

## 6 Challenges and future perspectives

### 6.1 From compositional variability to AI-driven precision fortification

A major challenge in functional egg development is the substantial variability in bioactive component deposition, arising from differences in feed formulation, hen physiology, production systems, and environmental conditions. This compositional heterogeneity not only hampers product standardization and reproducibility, but also complicates health claim substantiation, regulatory approval, and large-scale commercialization. In addition, enrichment strategies, particularly those involving polyunsaturated fatty acids, often face trade-offs between enrichment efficiency, oxidative stability, sensory quality, and cost-effectiveness. Recent advances in artificial intelligence-assisted feeding systems, digital nutrition management, and real-time physiological monitoring offer promising solutions to these limitations. AI-driven precision fortification could enable dynamic adjustment of nutrient inputs according to hen status, production stage, and targeted egg functionality, thereby improving deposition consistency while minimizing oxidative risk and unnecessary supplementation. Such approaches represent a critical shift from empirical fortification toward data-driven and mechanism-informed nutrient delivery.

### 6.2 From nutrient carriers to biologically active systems: the emerging “gut-egg axis”

Functional eggs have traditionally been regarded as passive carriers of enhanced nutrients. However, growing evidence indicates that maternal gut microbiota can modulate nutrient absorption, metabolic conversion, and subsequent deposition into eggs, giving rise to the concept of a “gut-egg axis.” While microbial variability introduces additional complexity and inter-individual variation in enrichment outcomes, it also presents new opportunities for targeted intervention. Microbiome-oriented strategies, including probiotics, prebiotics, fermented feeds, and dietary fiber modulation, may enhance the bioavailability, metabolic retention, and stability of egg-derived bioactive compounds. Incorporating gut microbiota considerations into enrichment strategies could therefore move functional eggs beyond simple nutrient accumulation toward biologically active systems with improved functional efficacy.

### 6.3 Toward personalized and mechanism-driven functional eggs

Future progress in functional egg development will likely require a transition from population-level fortification to mechanism-driven and personalized nutrition strategies tailored to specific life stages and health conditions, such as aging, pregnancy, and metabolic disorders. Advances in multi-omics technologies, including nutrigenomics, metabolomics, lipidomics, and microbiomics, provide powerful tools to elucidate the pathways governing nutrient deposition and egg bioactivity, identify responsive biomarkers, and link egg composition to health-relevant outcomes. Integrating multi-omics insights with precision feeding systems and consumer stratification may ultimately redefine functional eggs as targeted nutritional interventions rather than generalized fortified foods.

## 7 Conclusion

Functional eggs represent a cost-effective and biologically efficient platform for delivering essential nutrients within a familiar dietary matrix. Advances in nutrient enrichment strategies and mechanistic understanding of deposition pathways have strengthened their potential role in precision and preventive nutrition. Moving forward, the integration of AI-driven feeding technologies, microbiome-oriented interventions, and multi-omics-based mechanistic insights is expected to improve enrichment consistency, functional efficacy, and personalization. Collectively, these developments may transform functional eggs from broadly fortified products into targeted dietary interventions designed to meet specific nutritional and health demands.

## Glossary

ALA: Primarily α-linolenic acid
DHA: Docosahexaenoic acid
EPA: Eicosatetraenoic acid
FAT: Fatty acid translocase
FATP4: Fatty acid transport protein 4
FFAs: Free fatty acids
GPx: Peroxidase
HDL: High-density lipoprotein
LDL: Low-density lipoprotein
ROS: Reactive oxygen species
2-MAGs: 2-Monoacylglycerols
5-MTHF: 5-Methyltetrahydrofolate
TG: Triglycerides
TrxR: Thioredoxin reductase
PUFAs: Polyunsaturated fatty acids
ω-3 PUFAs: Omega-3 polyunsaturated fatty acids
VLDLR: Low-density lipoprotein receptor
VTG: Vitellogenin
Fe: Iron
Cr: Chromium
I: Iodine
K: Potassium
Mn: Manganese
Na: Sodium
Se: Selenium
Zn: Zinc
